# Chromosomal CGH identifies patients with a higher risk of relapse in neuroblastoma without *MYCN* amplification

**DOI:** 10.1038/sj.bjc.6603820

**Published:** 2007-06-19

**Authors:** G Schleiermacher, J Michon, I Huon, C Dubois d'Enghien, J Klijanienko, H Brisse, A Ribeiro, V Mosseri, H Rubie, C Munzer, C Thomas, D Valteau-Couanet, A Auvrignon, D Plantaz, O Delattre, J Couturier

**Affiliations:** 1 INSERM U830, Institut Curie, Paris, France; 2 Département d'Oncologie Pédiatrique, Institut Curie, Paris, France; 3 Service de Génétique Oncologique, Institut Curie, Paris, France; 4 Service de Pathologie, Institut Curie, Paris, France; 5 Département d'Imagerie Médicale, Institut Curie, Paris, France; 6 Service de Biostatistique, Institut Curie, Paris, France; 7 Unité d'Hemato-Oncologie, Hôpital des Enfants, Toulouse, France; 8 Unité d'Onco-Hématologie Pédiatrique, Hôpital de la Mère et de l'Enfant- CHU de Nantes, Nantes, France; 9 Département de Pédiatrie, Institut Gustave-Roussy, Villejuif, France; 10 Service d'Hématologie et d'Oncologie Pédiatrique, Hôpital Trousseau, Paris, France; 11 Département de Pédiatrie, CHU, Grenoble, France

**Keywords:** neuroblastoma, pangenomic analysis, CGH, prognosis

## Abstract

Whereas neuroblastoma (NB) with *MYCN* amplification presents a poor prognosis, no single marker allows to reliably predict outcome in tumours without *MYCN* amplification. We report here an extensive analysis of 147 NB samples at diagnosis, without *MYCN* amplification, by chromosomal comparative genomic hybridisation (CGH), providing a comprehensive overview of their genomic imbalances. Comparative genomic hybridisation profiles showed gains or losses of entire chromosomes (type 1) in 71 cases, whereas partial chromosome gains or losses (type 2), including gain involving 17q were observed in 68 cases. Atypical profiles were present in eight cases. A type 1 profile was observed more frequently in localised disease (*P*<0.0001), and in patients of less than 12 months at diagnosis (*P*<0.0001). A type 2 genomic profile was associated with a higher risk of relapse in the overall population (log-rank test; *P*<0.0001), but also in the subgroup of patients with localised disease (log-rank test, *P*=0.007). In multivariate analysis, the genomic profile was the strongest independent prognostic factor. In conclusion, the genomic profile is of prognostic impact in patients without *MYCN* amplification, making it a help in the management of low-stage NB. Further studies using higher-resolution CGH are needed to better characterise atypical genomic alterations.

Neuroblastoma (NB), the most frequent extracranial solid tumour of childhood, is characterised by a wide variability of its clinical course, with a possibility of cellular maturation or spontaneous tumour regression on the one hand, or aggressive clinical behaviour with rapid progression despite intensive therapeutic approaches on the other hand.

Clinical markers, such as stage and age at diagnosis, are insufficient to precisely predict outcome in all patients. This had led to the search for additional markers that might enable a more robust prognostic classification of NB (Brodeur *et al*, 1997, 2003; Maris and Matthay 1999; Maris, 2005). Several recurrent genetic alterations have been identified in NB. Amplification of the *MYCN* oncogene is observed in approximately 20% of cases and is clearly associated with a poor outcome (Seeger *et al*, 1985). Variations of the ploidy have also been described in NB, with near-triploidy associated with an excellent outcome, and diploidy/tetraploidy correlating with a poorer outcome (Look *et al*, 1991). Finally, a number of segmental chromosome alterations have been reported. Deletions of chromosome 1p, observed in approximately 25% of cases, and more recently deletions of 3p and 11q, thought to harbour yet unidentified tumour suppressor genes, have also been associated with a poor outcome (Caron *et al*, 1996; Schleiermacher *et al*, 1996; Luttikhuis *et al*, 2001; Attiyeh *et al*, 2005). Other recurrent chromosome losses involve chromosomes 4p, 9p, or 14q. Gain of chromosome 17q, harbouring hypothetical oncogenes, which might play a role in the oncogenesis of NB, represents the most frequent genetic abnormality in NB, and is thought to be a powerful independent predictor of poor outcome (Plantaz *et al*, 1997; Bown 2001; Brinkschmidt *et al*, 2001; Lastowska *et al*, 2001).

Until now, these various genetic markers are analysed separately by conventional karyotyping, 24-colour karyotyping, FISH and/or LOH studies (Ambros *et al*, 2003), making these analyses time consuming and rendering their interpretation difficult as not all markers could be studied at one time. During the last decade, pangenomic techniques such as comparative genomic hybridisation (CGH), which enable the analysis of the entire tumour genome in one step, identifying DNA copy number gains and losses across the whole karyotype, have been developed (Kallioniemi *et al*, 1992). The CGH technique constitutes a highly efficient and discriminative approach for the analysis of tumours characterised by quantitative genetic changes, which is the case in NB.

Chromosome CGH analysis of NB samples has enabled the identification of distinct genetic subtypes with specific prognostic characteristics (Brinkschmidt *et al*, 1997, 1998; Vandesompele *et al*, 1998, 2001; Lastowska *et al*, 2001; Plantaz *et al*, 2001). A genomic profile characterised by whole chromosome gains or losses, defining type 1 tumours (Lastowska *et al*, 2001), is observed more frequently in localised tumours and in children less than 1 year of age, with a good prognosis. On the other hand, unbalanced chromosome translocations leading to segmental chromosome gains or losses, including in particular chromosome 17q gain associated with either loss of 11q and 3p, or 1p (type 2 tumours), are observed more frequently in advanced stages of disease or older children, with an intermediate prognosis. Finally, tumours presenting *MYCN* amplification in addition to segmental chromosome alterations (type 3 tumours) show highly aggressive clinical behaviour and a poor outcome. It has been suggested that these subtypes represent distinct pathologies as transitions from one type to another appear to be rare.

A more recent study based on the pooled CGH data of 231 samples from several institutions has confirmed this classification (Vandesompele *et al*, 2005). Variations in the copy number of whole chromosomes was more frequently observed in localised disease, of favourable histology, and in infants, with recognition of a survivor signature conferring 100% 5-year survival in stage 1, 2 or 4s tumours presenting with whole chromosome 17 gain, whereas structural aberration patterns were a significant predictor of poorer outcome.

The interpretation of previous results may be hampered by the heterogeneity of techniques and by the inclusion of *MYCN* amplified tumours. The prognostic value of *MYCN* amplification is so strong that, in a clinical setting, the need for reliable genomic typing mainly concerns *MYCN* non-amplified tumours. To date, no study has focused specifically on the analysis by CGH of this subset of NB, so we report here the results of an extensive analysis by chromosomal CGH of 147 NB samples without *MYCN* amplification, in order to assess the clinical usefulness of this pangenomic technique for the genomic typing of these tumours.

## MATERIALS AND METHODS

### Patients

Patients with a histologically confirmed diagnosis of NB were included in this study, if frozen tumour material obtained at the time of diagnosis was available for DNA extraction. Only cases without *MYCN* amplification were included. Patients were treated in centres of the Société Française des Cancers de l'Enfant (SFCE) between 1990 and 2003, according to the ongoing national or international treatment protocols: for infants less than 1 year at diagnosis, NBL90 or INES99; for patients with localised resectable disease, LNESG1; for patients with localised unresectable disease, NB94 or NB02; for patients with metastatic disease, NB87ter or HR-NBL-01. Inclusion in the clinical trials was performed according to the relevant French law, following informed consent.

A total of 147 patients were included. The clinical characteristics of the patients corresponded to those described in NB in general (Brodeur, 2003). The median age of patients at diagnosis was 12 months (range 0–175 months). Median follow-up was 57.3 months (range 17–194 months). Tumours were stages 1 or 2 in 67 cases, stage 3 in 10 cases, stage 4 in 60 cases and stage 4S in 10 cases (Table 1a and b). There have been 50 relapses or disease progressions, of which eight occurred only at local sites, whereas 38 involved metastatic sites; the site of relapse was unknown in four cases. Of the 147 patients, 25 have died of disease, three have died of treatment toxicity and one of other cause, whereas the other 118 are alive (seven with progressive disease, two with stable disease, eight in partial remission, 101 in complete remission).

### Tumour samples

Tumour samples were obtained at the time of diagnosis by needle-core or surgical biopsy, primary surgery or fine-needle aspiration. For biopsies and surgical fragments, the tumour cell content was systematically checked on haematoxylin/eosin-stained frozen section of the sample submitted to DNA extraction. Fine-needle aspirates were checked by the cytologist on May–Grünwald–Giemsa-stained spreads. Only cases with more than 60% tumour cells were included (Ambros *et al*, 2003). The *MYCN* status was assessed by FISH using a *MYCN* probe (Zymed Laboratories, San Francisco, CA, USA) on frozen sections for tumour fragments, and on cytogenetic preparations for fine-needle aspirates. Tumours showing an average number of signals per nucleus >10 were considered as having an amplification, and were excluded from the study. Rare cases showing a borderline count of signals were checked with a centromeric probe of chromosome 2, and those showing a ratio *MYCN*/centromere signal >4 were considered as amplified and excluded. The whole tissue sections or cell spreads were thoroughly examined, in order to detect eventual heterogeneous amplifications (Ambros *et al*, 2001), but none was found.

### CGH analysis

Comparative genomic hybridisation was performed in one laboratory, as previously described (Kallioniemi *et al*, 1992). Briefly, tumour DNAs were labelled using a SpectrumGreen-dUTP (Vysis, Downers Grove, IL, USA) and a dedicated nick-translation kit (Vysis). SpectrumRed normal male DNA (Vysis) was used as reference. The hybridisation mixture was composed of 400 ng of tumour DNA, 400 ng of reference DNA and 80 *μ*g of Cot-1 DNA (Invitrogen, Cergy-Pontoise, France) in 15 *μ*l hybridisation buffer (formamide 50%/NaH_2_PO_4_ 40 mM/SDS 0.1%/dextran sulphate 10%/2X SSC). Comparative genomic hybridisation images of a minimum of 10 metaphases were captured with an epifluorescence Leica DMRB microscope fitted with a Photometrix CoolSnap fx digital camera, and analysed with Quips software (Vysis). For scoring chromosome imbalances, gains corresponded to green/red fluorescence ratios >1.2, and losses to ratios <0.8. Special attention was paid to the pattern of chromosome 1p imbalances, because of possible difficulties of interpretation described for this region (Kallioniemi *et al*, 1994). A loss was retained when the shift of the profile was clear and the green/red fluorescence ratio similar to that of other lost genomic regions, or, in case of ambiguous imbalance, when it was confirmed by FISH or array-CGH results. On the whole (94% of the cases), profiles of individual tumours could be classified into three genomic types, according to a typing adapted from Lastowska *et al* (2001): type 1, showing gains and losses of entire chromosomes; types 2, showing partial chromosome gains and losses of the chromosome regions known to be recurrently involved in NB (i.e., 1p, 2p, 3p, 11q and 17q), without (type 2a) or with (type 2b) 1p deletion, among other imbalances.

### Statistical analysis

Correlation between clinical and molecular data was assessed by using the *χ*^2^-test. Event-free survival (EFS) and overall survival (OS), indicated with the standard deviation, were estimated with the Kaplan–Meier method and compared by the log-rank test. A *P*-value of less than 0.05 was considered significant. Event-free survival was calculated from diagnosis until the date of last follow-up or event (tumour progression or relapse). Overall survival was calculated from diagnosis to the last follow-up or disease-related death. Multivariate analysis was conducted on EFS, using a Cox regression model, with a backward procedure.

## RESULTS

### CGH

**Figure 1:**
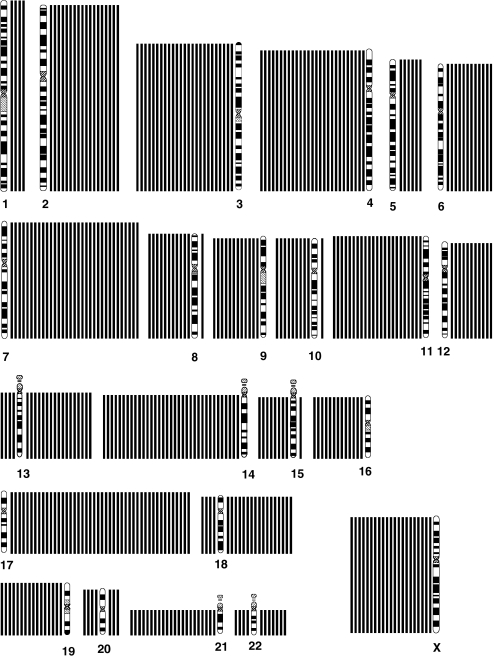
Diagram showing gains and losses detected by chromosomal CGH in tumours with a type 1 genomic profile, characterised by numerical chromosome alterations. Losses are indicated by a bar on the left and gains by a bar on the right of each chromosome ideogram. Each bar represents an alteration observed in one tumour.

**Figure 2:**
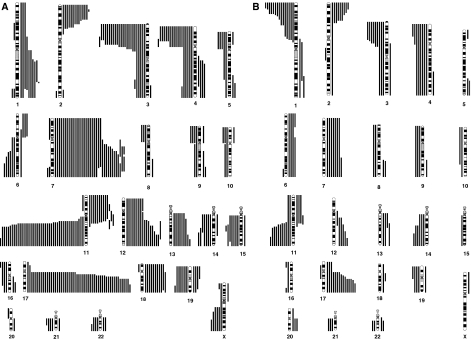
Diagram showing gains and losses detected by chromosomal CGH in tumours with a type 2 genomic profile, characterised by segmental chromosome alterations. Losses are indicated by a bar on the left and gains by a bar on the right of each chromosome ideogram. Each bar represents an alteration observed in one tumour. (**A**) Segmental alterations observed in tumours without chromosome 1p deletions (type 2a). (**B**) Segmental alterations observed in tumours with chromosome 1p deletions (type 2b).

Genomic typing using chromosomal CGH enabled the identification of distinct profiles. Comparative genomic hybridisation profiles showed only gains or losses of entire chromosomes in 71 cases (48% of all cases) (genomic type 1; Figure 1). Partial chromosome gains or losses, including gain of the chromosome 17q region, without or with deletion of chromosome 1p (type 2a or type 2b), were observed in 53 (36%) and 15 (10%) cases, respectively (Figure 2A and B; Table 1a). Among four cases showing a questionable 1p deletion, one was confirmed by the FISH result, the three other did not show any deletion by FISH and/or array-CGH, and were classified as type 2a. Eight additional tumours could not be classified into these genomic types, and were considered as having an atypical profile.

#### Genomic type 1

In these tumours, the most frequent imbalance was whole chromosome 17 gain, observed in 77% of the cases, followed by −14, +7, −4, −3, −11, −21, +2, +13, +18, −19, +5, +6, −16, each present in more than 20% of cases.

#### Genomic types 2

Types 2a and 2b represented 78 and 22% of all genomic type 2 cases, respectively. All of these tumours showed 17q gain, except one type 2a and three 2b tumours, having a gain of an entire chromosome 17, but associated with typical segmental imbalances of other chromosomes. Main segmental imbalances other than 17q gain were 11q and 3p deletions (78 and 37% of type 2 cases, respectively), 2p gain (32%) and 4p deletion (22%). Among the 53 type 2a tumours, 17 cases had deletions of both 3p and 11q, two had deletion of 3p without deletion of 11q, 26 had deletion of 11q without deletion of 3p and eight did not have either of these aberrations. Among the 15 type 2b cases, in addition to chromosome 1p deletion, six also had deletion of both 3p and 11q, whereas three others had deletion of 11q without deletion of 3p. Some of these cases also showed few whole chromosome imbalances, involving mainly gains of chromosomes 7, 12, 13 and 18, in addition to the segmental changes.

#### Atypical genomic types

For four tumours, no genomic alterations could be identified, despite sufficient tumour cell content of the sample and confirmation of the histological diagnosis. In three other cases, segmental alterations not typically observed in NB were observed (case 93: −19p, −22; case 94: −11q14q25, −14q21q32 without chromosome 17q gain; case 142: +17p11.2q25 without segmental loss). For a last case (case 41), two samples from the same tumour had been received from the pathologist and were separately analysed. A 1p loss was present in addition to other segmental abnormalities in one of the samples only, so the tumour could not be assigned to 2a or 2b type, and was classified into the atypical group.

### Clinical characteristics according to the genetic subtypes

The clinical characteristics in the different genetic subgroups are presented in Table 1a and b. The distribution of frequency of the different variables was not random (Table 1b). A genomic type 1 profile was observed more frequently in localised disease (stages 1–3; *P*< 0.0001), and in patients of less than 12 months at diagnosis (*P*< 0.0001), whereas type 2a/2b profiles occurred more frequently in stage 4 disease, in patients older than 12 months, and in tumours located in the abdomen.

### Univariate survival analysis

**Figure 3:**
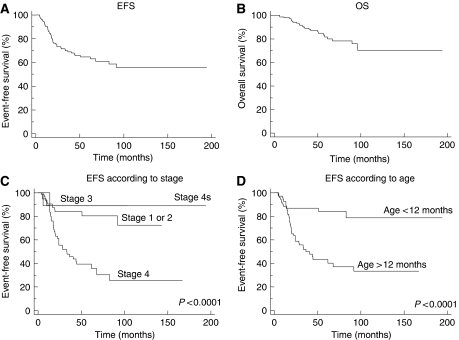
Survival curves of 139 neuroblastoma patients. (**A**) Event-free survival of all patients. (**B**) Overall survival of all patients. (**C**) Event-free survival according to stage at diagnosis. (**D**) Event-free survival according to age at diagnosis.

The eight tumours with atypical profiles were excluded from the survival analysis. Among the 139 remaining patients, EFS at 4 years was 65.9±4.2 % and 4-year OS was 87.1±3.0% (Table 2; Figure 3A and B). As expected, patients with stage 4 disease and those aged over 12 months at diagnosis had a poorer EFS and OS (*P*< 0.0001; Figure 3C and D). Patients with an abdominal primary had a poorer OS (*P*< 0.04).

**Figure 4:**
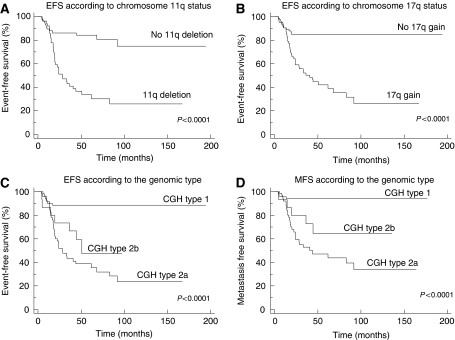
Survival curves according to genetic markers. (**A**) Event-free survival according to chromosome 11q status. (**B**) Event-free survival according to chromosome 17q status. (**C**) Event-free survival according to the genomic profile, type 1 referring to numerical alterations, and type 2a and 2b referring to structural chromosome alterations without or with chromosome 1p loss. (**D**) Metastasis-free survival according to the genomic profile.

Single genetic markers, including chromosome 3p and 11q deletions and chromosome 2p and 17q gains, were all associated with a significantly poorer EFS and OS (Figure 4A and B). Chromosome 1p deletion was not associated with statistically significant poorer EFS and OS.

In addition to the single genetic markers, the genomic type also proved to be of prognostic significance. Indeed, patients with a type 1 genomic profile had a significantly better EFS and OS (Figure 4C). In the entire population, only one patient with a type 1 CGH profile has died. This patient, aged 2 years, had metastatic disease at diagnosis and subsequent metastatic relapse.

Comparative genomic hybridisation profiles of type 2a and 2b were associated with a higher risk of relapse or progression in the overall population, and with a poorer outcome (log-rank test, *P*< 0.0001). No difference in EFS or OS between CGH types 2a and 2b could be observed (log-rank test; *P*=0.2 and *P*=0.8, respectively). Among patients with type 2a or 2b tumours, those with 11q deletion did not have a higher risk of relapse than those without chromosome 11q deletion (log-rank test; *P*=0.14), but did have a poorer overall survival (log-rank test; *P*=0.03).

In NB, local recurrences can often be successfully treated, whereas metastatic relapse still represents a major challenge. We therefore analysed the impact of the genomic profile on metastatic free survival. Type 2a and 2b genomic profiles were associated with a significantly poorer metastatic free survival in the overall population (Figure 4D).

### Survival analysis in patients with localised disease

Whereas in 66 patients with metastatic disease, 33 of 36 relapses occurred at a metastatic level, among the 73 patients with localised disease, over half of the relapses consisted of purely local relapses (8/13, 61%; Table 3). Only four patients with initially localised disease had metastatic relapse, and, of these, two had a type 2a or 2b genomic profile. The tumours of the other two patients with a type 1 genomic profile will certainly merit further investigation. Among patients with localised disease, those with a type 2a or type 2b profile had a significantly higher risk of relapse when compared to those with a type 1 profile (log-rank test; *P*=0.005).

### Regression model

In a final step, multivariate analysis using a regression model on EFS, progressively removing variables in case of *P*>0.05, was performed, according to a Cox model. The variables age, stage, status of 1p, 2p, 3p, 11q and 17q, as well as the CGH profile, were used in the model. In this model, the CGH profile (type 2a or 2b *vs* type 1) was the strongest independent prognostic factor (RR 4.4; *P*=0.0004; Table 4).

## DISCUSSION

Neuroblastic tumours without *MYCN* amplification are characterised by combinations of non-random genomic alterations consisting of numerical chromosome variations on the one hand, and segmental chromosome imbalances related to unbalanced translocations on the other hand. Chromosome CGH, and more recently array-CGH, are techniques of choice for assessing such imbalances in a single experiment (Kallioniemi *et al*, 1992). To date, several series of CGH analyses of non-selected NBs including *MYCN* amplified cases have been published, mostly consisting of data from several centres (Brinkschmidt *et al*, 1997; Lastowska *et al*, 2001; Vandesompele *et al*, 2005). These previous analyses have shown that, using CGH, tumours could be classified into genomic groups associated with distinct clinical characteristics. We have now performed chromosomal CGH of NB samples obtained from centres of the SFCE in a single laboratory, in order to determine if pangenomic genotyping could be useful for the management of *MYCN* non-amplified tumours in a clinical setting.

Our study clearly shows that pangenomic analysis enables the determination of distinct genomic profiles. To date, no common international consensus nomenclature exists for the description of the different genomic types, and we have adapted the previously published categories (Lastowska *et al*, 2001). Among type 1 profiles, alterations, by order of frequency, were gains of chromosomes 17, 7, 2, 13 and 18, and losses of chromosomes 14, 4, 3, 11, 19 and 21, consistent with previous reports. Among tumours with partial chromosome imbalances, thought to arise from unbalanced translocations, gains of chromosome 17q were observed most frequently, as reported previously. Other recurrent segmental imbalances were deletions of 11q, 3p or 4p and gain of 2p. Taking into account the clinical importance given until now to 1p status, we have, as a first approach, divided type 2 tumours into subtype 2a (normal chromosome 1) and subtype 2b (presence of 1p deletion). The majority of all samples could be classified according to these criteria (139/147). Only eight cases could not be classified; four did not have any genomic imbalance detected by CGH, despite an adequate tumour cell content of the sample. This may possibly be explained by the presence of very small segmental alterations below the resolution of CGH, or by a purely triploid chromosome number. Finally, four other tumours showed segmental chromosome gains or losses in an atypical pattern.

The resolution of chromosomal CGH of one sub-band, or approximately 10 Mb, does not allow the analysis of chromosome breakpoints in detail. Whereas breakpoints are widely dispersed along chromosome arms 1p, 2p, 3p and 17q, they lie closer together on chromosome 11q, at 11q23, in accordance with published data (Schleiermacher *et al*, 2004; Stallings *et al*, 2004; White *et al*, 2005; Spitz *et al*, 2006). Further studies using FISH or high-resolution CGH will be needed to map the breakpoints precisely.

A large number of studies have shown an association between genetic markers and clinical characteristics. Indeed, it has been shown that the presence of structural abnormalities due to unbalanced chromosome translocations is clearly associated with advanced stages of disease (Lastowska *et al*, 2001; Bilke *et al*, 2005; Vandesompele *et al*, 2005). These unbalanced translocations most frequently involve chromosome 17q as the donor and chromosomes 1p, 3p or 11q, amongst others, as the recipient chromosome, leading to genomic imbalance. The most frequent genomic imbalance observed in high risk NB is chromosome 17q gain (Lastowska *et al*, 1997; Bown *et al*, 1999), with other copy number changes involving chromosome 11q loss, often associated with chromosome 3p loss, in the absence of 1p loss (Breen *et al*, 2000; Plantaz *et al*, 2001). In this study, a clear association between the presence of genomic imbalances, as in type 2a and type 2b tumours, and advanced stage of disease is also observed. An association between deletion of chromosome 11q and 3p is also observed. However, the analysis of type 2b profiles shows that 1p and 11q deletions are not exclusive. On the other hand, previous studies have shown that localised tumours or those occurring in infants <1 year of age have numerical rather than structural abnormalities (Bilke *et al*, 2005; Vandesompele *et al*, 2005). In our series, a type 1 genomic profile is also associated with an age <1 year at diagnosis and with localised disease. It has also been shown that in stage 4s tumours, structural abnormalities are observed in cases with aggressive clinical behaviour, whereas spontaneously regressing tumours more frequently have numerical aberrations (Brinkschmidt *et al*, 1998).

In this study, established clinical factors were of prognostic significance, such as tumour stage and age at diagnosis. We used an age cut-off of 12 months, rather than the age of 18 months suggested recently (London *et al*, 2005), as the treatment protocols according to which patients were treated were based on the 12-month cut-off. Furthermore, nearly all genetic markers have been shown to be of prognostic significance in univariate analysis. Chromosome 17q gain, the most frequent genetic alteration in NB, is associated with a poor outcome (Abel *et al*, 1999; Bown *et al*, 1999; Brinkschmidt *et al*, 2001). More recently, a study looking at 1p and 11q status by determining LOH has shown a strong prognostic impact of imbalanced 11q loss (Attiyeh *et al*, 2005). Chromosome 1p and 3p loss, as well as 2p gain, have also been associated with a higher risk of relapse (Caron *et al*, 1996; Ejeskar *et al*, 1998; Maris and Matthay, 1999; Spitz *et al*, 2003, 2006). In this study, consistent with these previous observations, single genetic markers, including loss of 3p and 11q and gain of 2p and 17q, are all strong predictors of poor outcome. Deletion of 1p is not associated here with a poor outcome in univariate analysis in the overall population. However, patients with a 1p deletion did have higher risk of relapse when compared to only those with a type 1 genomic profile. These results indicate that 1p deletion is a marker, which identifies only a small subgroup of patients at risk in the global population.

To date, few studies have focused on the prognostic impact of these various genetic markers in a multivariate setting, especially in *MYCN* non amplified tumours. Taking into account the well-known prognostic value of *MYCN* amplification, the only genetic marker retained in clinical protocols in Europe at present, pertinent genomic stratification is especially needed in *MYCN* non amplified low stage and infant tumours. Our study now suggests that the presence of any of the segmental alterations typically observed in NB, grouped together in the type 2 genomic profile, is strongly associated with a higher risk of relapse, a higher risk of metastatic relapse, as well as a poorer outcome in the overall population. Furthermore, a genomic type 2 profile is associated with a higher risk of relapse, and a higher risk of metastatic relapse among the patients with localised disease. The findings of this study are in accordance with a recent report indicating that structural abnormalities of chromosomes 1p, 3p, 11q and 17q are observed more frequently in tumours with recurrence (Spitz *et al*, 2006). In this study, among patients with localised disease, only two with a type 1 genomic profile had metastatic relapse. These tumours certainly merit further analysis using high resolution CGH in order to search for small segmental alterations not detected by chromosomal CGH. Our data indicate that maximal prognostic information can be obtained when looking at the entire genomic profile. Furthermore, detailed analysis using higher-resolution array-CGH of a more extensive series of samples is needed to answer the question if the atypical profiles harbouring segmental alterations not typically observed in NB may also be associated with a higher risk. More detailed pangenomic analyses with higher resolution are required to perform a better genomic classification of tumours, to advance in the classification of atypical profiles, and to determine a common nomenclature.

Genomic profiles provide a comprehensive overview of genomic changes in NB and are of prognostic impact in patients without *MYCN* amplification, making them a help in the management specifically of low stage tumours. Indeed, treatment desescalation could possibly be proposed in future clinical trials for some patient subgroups, as for instance for infants with asymptomatic, non-resectable low-stage tumours. In case of a type 1 genomic profile, the feasibility of an observational attitude could be investigated, whereas patients with type 2 tumours could be excluded from treatment desescalation. On the other hand, more intensive therapy could be discussed for other patient subgroups, such as older patients with a localised but unresectable tumour, with a type 2 profile.

In conclusion, the genomic profile is thought to be representative of an underlying genomic abnormality. In tumours with numerical aberrations, an abnormality in the mitotic segregation of the chromosomes is thought to exist. On the other hand, the structural chromosome alterations in high- and intermediate-risk tumours are most frequently due to unbalanced chromosome translocations, which in turn are thought to arise from DNA double-strand breaks repaired erroneously. A DNA maintenance or repair pathway is most likely impaired. We have recently suggested that the DNA repair mechanism leading to the DNA double strand-breaks is most likely break-induced replication (Janoueix-Lerosey *et al*, 2005; Schleiermacher *et al*, 2005). Thus, the single genetic alterations could be considered as surrogate markers for an underlying abnormality, which will confer additional selective advantage to tumour cells. This study suggests that a pangenomic analysis should be performed for all NB at diagnosis for a better understanding of the oncogenesis of NB and for better therapeutic stratification.

## Figures and Tables

**Table 1a:** Genetic markers according to genomic profiles^a^

		**CGH type**			
**Genetic marker**		**1 (***n***=71)**	**2a (***n***=53)**	**2b (***n***=15)**	**Atypical (***n***=8)**
Chr 1p	No deletion	71	53	0	8
	Deletion	0	0	15	0
Chr 2p	No gain	71	39	7	8
	Gain	0	14	8	0
Chr 3p	No deletion	71	34	9	8
	Deletion	0	19	6	0
Chr 11q	No deletion	71	10	6	7
	Deletion	0	43	9	1
Chr 17q	No gain	71	1	3	6
	Gain	0	52	12	2

Abbreviation: CGH, comparative genomic hybridisation.

a Taking into account only partial chromosome imbalances.

**Table 1b:** Clinical characteristics according to genomic profiles

		**CGH type**				
**Clinical characteristics**		**1**	**2a**	**2b**	**Atypical**	***P***^a^(*χ*^**2**^**-test)**
Stage (INSS)	1 or 2	50	11	3	3	
	3	7	2	0	1	
	4	5	40	12	3	
	4s	9	0	0	1	<0.0001
Age (months)	⩽12	54	9	7	4	
	>12	17	44	8	4	<0.0001
Localisation	Abdominal	40	43	12	5	
	Extra-abdominal	19	6	3	3	
	Not known	12	4	0	0	<0.05

Abbreviation: CGH, comparative genomic hybridisation.

a Atypical cases excluded.

**Table 2:** EFS and OS according to clinical and genetic markers

**Marker**		**4-year EFS (%)±s.e.**	***P* (log rank)**	**4-year OS (%)±s.e.**	***P* (log rank)**
Entire population		65.9±4.2		87.1±3.0	
					
Age at diagnosis	⩽12 months	87±4.1		100	
	>12 months	43.4±6.6	<0.0001	73.3±5.8	<0.0001
Stage	1,2,3 or 4s	84.6±4.3		100	
	4	45.8±6.5	<0.0001	69±6.5	<0.0001
Tumour localisation	Extra-abdominal	78.6±7.8		96.3±3.6	
	Abdominal	61.1±5.3	=0.08	83.5±4.1	<0.04
					
Chromosome 1p	No deletion	67.0±4.4		87.9±3.2	
	Deletion	59.3±12.9	NS	80±10.3	NS
Chromosome 2p	No gain	72.1±4.3		90.3±2.9	
	Gain	33.1±10.9	=0.0001	68.6±10.7	=0.0002
Chromosome 3p	No deletion	72.5±4.4		92.1±2.7	
	Deletion	36.5±10	<0.0001	64±10.4	=0.0002
Chromosome 11q	No deletion	84.2±4.1		98.8±1.2	
	Deletion	36.6±6.9	<0.0001	68.3±6.8	<0.0001
Chromosome 17q	No gain	85±4.2		97.2±2	
	Gain	44.7±6.5	<0.0001	75.9±5.7	<0.0001
CGH type	Type 1	88.6±3.8		98.6±1.4	
	Type 2a or 2b	43.5±6.3	<0.0001	75.8±5.5	<0.0001

Abbreviations: CGH, comparative genomic hybridisation; EFS, event-free survival; OS, overall survival.

**Table 3:** Frequency and site of relapse according to the CGH profile

	**Type of relapse**		
**Stage of disease**	**CGH type 1 (*n*=71)**	**CGH type 2a (*n*=53)**	**CGH type 2b (*n*=15)**
Localised disease	Local: 3	Local: 3	Local: 2
(stage 1, 2 or 3)	Metastatic: 2	Metastatic: 2	
(*n*=73)	Unknown: 1		
Metastatic disease	Local: 0	Local: 0	Local: 0
(stage 4 or 4s)	Metastatic: 2	Metastatic: 26	Metastatic: 5
(*n*=66)		Unknown: 3	

Abbreviation: CGH, comparative genomic hybridisation.

**Table 4:** Cox proportional hazard model (EFS; backward model)

**Variable**	**RR**	**95% CI**	***P*-value**
*CGH type*			
Type 1	1		
Type 2a or 2b	4.62	2.03–10.5	0.0003
			
*Age*			
⩽12 months	1		
>12 months	2.20	1.06–4.58	0.034
			
Chr 1 p status	—	—	NS
Chr 2p Status	—	—	NS
Chr 3p status	—	—	NS
Chr 11q status	—	—	NS
Chr 17q status	—	—	NS
Stage	—	—	NS

Abbreviations: CGH, comparative genomic hybridisation; CI, confidence interval; RR, relative risk.
